# The Role of Metacognitive Skills in Music Learning and Performing: Theoretical Features and Educational Implications

**DOI:** 10.3389/fpsyg.2019.01583

**Published:** 2019-07-12

**Authors:** Eleonora Concina

**Affiliations:** Department of Philosophy, Sociology, Education and Applied Psychology, University of Padova, Padua, Italy

**Keywords:** music, performance, metacognition, self-regulation, music learning

## Abstract

Metacognition is a key component of musical performance. Metacognitive knowledge and skills are fundamental for musicians at all stages of their academic and professional career to allow them to structure, monitor, assess and, if needed, revise practice sessions toward specific performance goals. Research in music education has highlighted the impact that metacognition has on enhancing musical performance and the learning processes that characterize it. Expert musicians usually show a high level of metacognitive competence, which allows them to effectively self-regulate their learning activity while preparing for a performance. While professional musicians seem to have a wide range of learning strategies and skills, music students are not always aware of the importance of adopting a metacognitive approach in their learning process. In addition, the metacognitive dimension is not always explicitly addressed during music lessons, leaving students to adopt inefficacious learning strategies, or to repeatedly use sets of strategies in an incorrect manner. The aim of the current paper is to present and discuss the most recent studies on metacognition in music performance from an educational perspective that focuses on process as well as results. The role of metacognitive competence in musical activity will be discussed, first by examining the components of the metacognitive competence exhibited by expert musicians, and then by focusing on the impact of metacognition on music students’ learning. Educational implications for enhancing the learning experience of young musicians will also be discussed.

## Introduction

Listening to a highly accomplished musician could lead non-musicians and novice players to believe that making music is a simple and easy task. In reality, for all music genres, music making is a complex, and highly structured activity which requires musicians to plan and manage several specific skills (cognitive, emotional, motivational, and strategical). These characteristics of musical performance require not only the effort of continuous learning, but also the search for increasingly effective practice strategies. The final outcome of music performance relies heavily on what goes on during practicing, since successful performances require a great effort for learning and refining musical pieces, setting performance outcomes, planning effective practice sessions, and allocating time and personal resources for structuring them. To reach their goals, performers must be able to self-organize, direct, and assess their musical learning activity through practice (McPherson and Zimmerman, 2011). This, in turn, implies the acquisition of knowledge and skills concerning how the learning process can be optimized in terms of cognitive, emotional, and motivational factors. Metacognition has been broadly defined as knowledge and awareness about cognition and cognitive processes (Flavell, 1979). This definition also includes the ability to organize, monitor, and regulate those processes with the aim of achieving specific learning outcomes (Pintrich et al., 2000). The metacognitive dimension includes all the abilities and information needed to manage the executive strategies involved in music performance: it is fundamental for organizing musical practice, directing individual choices and efforts toward specific strategies, and promoting the effective management and monitoring of time and personal resources.

Enhancing musical practice means not only spending more time playing or singing but also using metacognitive skills to engage in effective practice sessions (Bathgate et al., 2012). Although often little considered, metacognitive competence is also a key factor for critically self-evaluating one’s performance (Hart, 2014); that is, responding competently to personal successes and failures to improve practice, and achieve learning goals. The adoption of a metacognitive approach in music learning allows musicians to learn more easily by optimizing the time dedicated to practice and improving the memorization and retention of the musical material learned (Hart, 2014). Current research in music education is focused on examining the metacognitive dimension of music learning. This research interest is supported by a new pedagogical perspective that considers the processes of learning and practicing just as important as performance outcomes for evaluating and understanding success and failure in music learning (Leon-Guerrero, 2008; Wise et al., 2017). The aim of the current paper is to critically discuss the role of metacognition in music performance and the development of metacognitive skills in music learning, making references to the most recent research about these topics in the context of Western classical music. By considering learning as a process, educational implications for music education will also be discussed. In the first section the features of the general concept of metacognition will be presented, with reference to the main findings in the educational research. In the second section, the role of the metacognitive components in music performance will be introduced and discussed. Their main characteristics will be examined as they appear in the activity of expert musicians, and how they can be achieved will be considered in relation to research on beginner and middle-level music students. The last section will focus on the main educational implications for promoting the development of a metacognitive competence in musical practice and performance.

## An Overview of Metacognition and Its Correlates

In the music domain, the metacognitive dimension plays an important role in learning activity because it allows for planning, regulating, monitoring, and assessing cognitive processes and their results. Indeed, metacognitive competence has a key role in the learning process in all the knowledge domains. For this reason, metacognition has been widely studied in educational research (Flavell and Wellman, 1975; Flavell, 1979; Davidson and Sternberg, 1998; Pintrich et al., 2000), with particular emphasis on its impact on academic learning processes and outcomes. In addition, more recent studies have focused on the relationship between the development of metacognitive knowledge and skills and the improvement of learning expertise (Pressley et al., 1987; Schraw, 1998; Sternberg, 1998). Finally, several research contributions have examined the relationship between self-regulation and metacognition using different approaches (Zimmerman, 1995; Pintrich et al., 2000).

Cognition is a complex construct: it involves human mental activity that expresses itself in many different cognitive tasks (e.g., making plans, categorizing, learning, and using language, etc.; Bechtel et al., 1998). It is closely related to and influenced by the emotional, affective, and motivational dimensions of knowing (Gardner, 1985; Thompson, 2007). More recent theories have developed an innovative perspective of “embodied cognition,” which has reconsidered the role of experience and the impact of the environment on human cognition (Wilson, 2002). According to this, many mental states, intuitions, and thoughts are affected by the interrelated work of mind and body in experiencing the external world. This framework also introduces the need to take into account both the actions and reflections made by the individual in managing the cognitive activity. From this perspective, metacognition and cognition are closely interrelated: while cognition is characterized by all the knowledge and the abilities related to a specific field, metacognition can be considered the “high-order agent” [as defined by Veenman et al. (2006), pp. 5] which allows one to plan, monitor, guide, assess, and revise the cognitive activity. Music education has also drawn on the concept of metacognition as high-order activity (Benton, 2014), examining how expert musicians plan, monitor, and regulate their own behaviors in order to fulfill performing tasks.

The earliest sustained study of metacognition dates to the work of Flavell and Wellman (1975), which focused specifically on the metacognitive dimension related to memory, called metamemory. Metamemory includes all of a person’s knowledge about the memory process and its phases as well as the strategies used to facilitate and improve memorization, retrieval, and recall (Flavell and Wellman, 1975). Understood in this way, metamemory is a component of a more general metacognitive dimension that affects the learning process and enhances its strategic features. More specifically, Flavell (1979) defined metacognition as a cognitive monitoring process characterized by four main aspects: metacognitive knowledge, metacognitive experiences, individual goals, and strategies. All these elements are inter-related, and they evolve continuously throughout life, contributing to the ability to self-regulate one’s learning processes in all the knowledge domains. More recently, research findings have highlighted that the integration of knowledge and skills is a key feature of metacognition. In a metacognitive approach, it is necessary not only to be aware of the different phases and characteristics of the learning process and strategies that can be applied, but it is also important to put these strategies into action and to monitor, and reflect critically upon the current educational experience. For example, a student may know that making a written summary of the assigned material, highlighting its core features (e.g., key concepts, important dates, etc.), could be useful for better understanding and learning it. At the same time, he or she has also to be aware of the characteristics of a good summary (metacognitive knowledge), and what should be done for obtaining it from the source material (metacognitive skills and application of strategies). This is also particularly true in musical practice, where the musician has to select and apply learning strategies considering both personal, and situational aspects (e.g., goals, material to be learned, available resources, etc.). Pintrich et al. (2000) recognized three main features of metacognition:

(1) Metacognitive knowledge (i.e., knowledge about knowledge).
(2) Metacognitive evaluation and monitoring (i.e., assessing and revising the learning process).
(3) Abilities to self-regulate cognitive processes (i.e., skills needed for self-organizing and managing cognitive processes).

The first feature has also been identified by Flavell (1979), while the second can be considered as an extension of the concept of metacognitive experiences. Such experiences can be examined and assessed, in order to gain awareness for enhancing cognitive processes. Finally, the self-regulation abilities include both the definition of learning goals and the selection and use of strategies.

**FIGURE 1:**
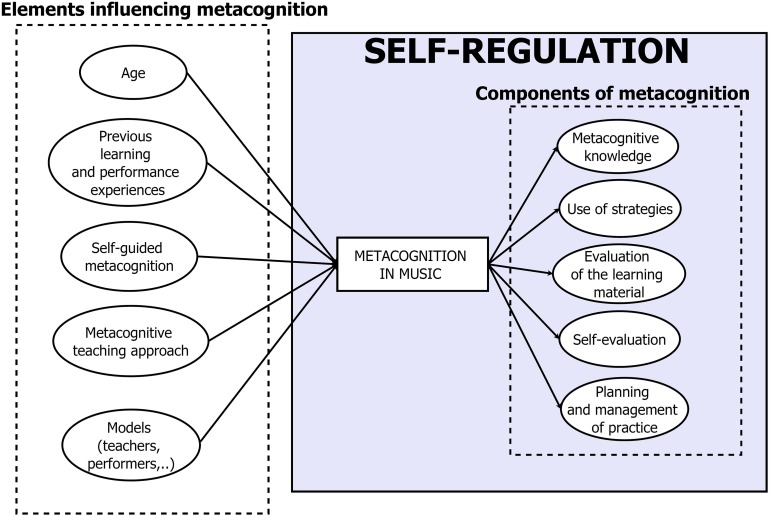
Schematization of the main aspects affecting metacognition in music and its components.

These are general aspects that characterize learning processes in all cognitive domains; however, there are some metacognitive features that can be considered domain-specific (Davidson and Sternberg, 1998) and are related to the intrinsic characteristics of the discipline considered. For example, the suitability and effectiveness of learning strategies depend on the domain in which they may be applied because they are mainly connected with the learning goals and the tasks defined for this specific area of knowledge. Anyway, for all the knowledge domains, a common feature that has a relevant impact on metacognitive competence is represented by the level of learner’s expertise. This is also true for music performance, where expert musicians exhibit high-metacognitive attitudes and behaviors (see Figure 1).

### Expertise

In general, in all the cognitive areas, metacognition seems to be an essential component of expertise (Sternberg, 1998). Advanced students and professionals can select the most suitable strategies, understand the level of difficulty and the possible challenges of a task, monitor their performance, and effectively allocate time resources. They can reach successful results by optimizing their efforts in the learning activity. Unlike novice learners, expert learners in all the knowledge domains are more aware of the relationship between performance outcomes and metacognition, and more able to use strategies effectively (Pressley et al., 1987). More specifically, they know many different strategies and are aware of how and when to adopt them during their learning activity to enhance the cognitive process and reach a positive outcome. Another aspect of expertise is characterized by flexibility in revising, adapting, and changing individual action plans in the face of challenging situations (Downing et al., 2006). In addition, expert learners usually adopt a growth mind set toward learning (Dweck, 2000), whereby personal effort and engagement in learning tasks are seen as integral to the development and enhancement of individual skills. A growth mind set is in contrast to the view that considers individual ability as fixed and unchangeable. From an educational perspective, the relationship between metacognition and expertise seems to be characterized by a gradual process of transfer of learning. If, at first, students learn metacognitive strategies within a specific cognitive domain, then, step by step, they become more flexible in using them and can transfer their metacognitive competence to other learning areas (Schraw, 1998). Educators may have a key role in promoting this process, for example by using a problem-based learning approach that encourages students to apply what they learn to other practical situations beyond the specific learning activity (Downing et al., 2006).

### Self-Regulation

From a perspective of self-regulated learning, metacognitive abilities are needed for self-control, planning, and monitoring one’s learning activity. Research has also investigated the relationship between metacognition and self-regulation. Many studies have recognized that metacognition is a core component of self-regulation in learning (Zimmerman, 1995), and the intercorrelations between these two constructs have been considered and examined. This educational process is characterized by an active learner who is autonomous in organizing and putting his or her learning process into action. Metacognition in the context of self-regulated learning includes both knowledge about cognition and the self, as well as about one’s ability to monitor and control their learning (Pintrich et al., 2000). It also depends on some other sociopsychological factors (Zimmerman, 1995), such as motivation, self-efficacy, self-esteem, and the causes to which people attribute their successes and failures (attributional theory), which can affect, positively, or adversely, the self-management of the learning process. In music performance, metacognition plays an important role: musicians set specific performance goals, and to reach these goals they must self-regulate their practice and learning processes.

## Metacognition and Self-Regulated Learning in Music Performance

Because musical activity may be both an individual and a collaborative task, metacognition can be recognized as a specific sub-competence of individual learning and a sub-dimension of social interaction and collaborative learning (Benton, 2014). Metacognition in musical performance is characterized by several components related to knowledge about the cognitive process and learning strategies (Nielsen, 1999; Hallam, 2001; Abushanab and Bishara, 2013), the abilities needed for effectively selecting and using these strategies for learning and memorizing the musical material (Nielsen, 1999; Ginsborg, 2002; Lisboa et al., 2015), and the ability to evaluate individual musical practice, learning outcomes and performance (Peynircioglu et al., 2014). All these features are summarized by the three dimensions proposed by Benton (2014) for metacognition in music performance, namely reflecting upon the task and the individual cognitive process (self-reflection), regulating individual activity (self-regulation), and evaluating individual performance (self-evaluation). Self-reflection includes thinking about the cognitive processes that the musician has activated during musical practice and performance. For example, asking oneself if the application of a specific learning strategy has led to the desired learning outcomes. Self-regulation is related to the act of giving self-instructions and directions for guiding individual practice. For example, when confronted by a challenging passage, planning how to study it for improving its interpretation. Self-evaluation refers to the assessment of the personal cognitive process. Usually, it is a key task that should be accomplished after each performance in order to identify strong points and critical issues.

Interest in metacognition in music learning has recently been supported by innovative educational theories. Among these, a notable contribution is that of “action-first” educational philosophies (Bowman, 2004; Elliot and Silverman, 2015). These advocate a new approach to music education which integrates the esthetic and emotional dimensions of music making with the rational strands of human cognition. In this holistic perspective that connects emotion and cognition, the role of cognitive and metacognitive features in music education has been reconsidered, and specifically addressed in musical pedagogy. This approach highlights the need to enhance students’ musical learning processes, in terms of empowering cognitive skills, and metacognitive competence for reaching successful learning outcomes in the musical domain.

Two main research questions have guided studies about metacognition in musical performance: (1) what are the main components of music metacognition, and (2) how can these components be developed during musicians’ formal training and professional career. With reference to the first question, the practice and performance of expert musicians have been considered, and analyzed. Concerning the second question, the research focuses specifically on the metacognitive skills exhibited by beginner, and middle level students (young and adult). In the discussion that follows, each metacognitive component will be presented first examining its main features as they have been measured in expert musicians’ activity, then discussing how it can be developed, and achieved by students during their training. The components of metacognition that have been examined in music education studies are included in Figure 1. Table 1 summarizes the main literature contributions to this research topic, distinguishing between studies on experts, and students of different levels.

**TABLE 1:** Summary of the main literature contributions in the research topic of music and metacognition.

**Metacognitive components**	**For expert musicians**	**For beginning music students**
1. Knowing and selecting strategies for musical practice	– Nielsen, 1999 – Hallam, 2001	– Nielsen, 2004 – Leon-Guerrero, 2008 – Bathgate et al., 2012 – Abushanab and Bishara, 2013

2. Organizing musical practice and evaluating musical material and performance outcomes	– Ginsborg, 2002 – Williamon and Valentine, 2002 – McPherson and Renwick, 2011 – Peynircioglu et al.,2014	– Bauer, 2008 – Benton, 2013 – Lisboa et al., 2015

3. Enhanced self-regulation during practice through metacognitive skills	– Jørgensen, 2004 – McPherson and Zimmerman, 2011 – Benton, 2014 – Araújo, 2016 – Varela et al., 2016 – Williamon et al., 2017	– Bartolome, 2009 – Hart, 2014 – Power and Powell, 2018

4. Managing self-regulation in collaborative practice and performance	– Gaunt and Westerlund, 2013 – Hanken, 2016 – Nielsen et al., 2018 – Schiavio et al., 2018 – Schiavio et al., 2019	– Benton, 2014 – Hanken, 2016

### Knowing and Selecting Appropriate Strategies in Musical Practice

Metacognitive skills allow reflection upon individual practice, and metacognitive knowledge is fundamental for organizing and directing strategic behaviors during musical practice (Miksza, 2012). When considering a specific learning goal, expert musicians can plan their practice sessions, selecting specific learning strategies in accordance with the different phases of learning. In general, the learning approaches are characterized by great variability and reflect a personalization of strategic patterns. According to Nielsen (1999), strategic behaviors in expert musicians’ practice may have different functions, such as categorizing the learning material, identifying critical passages of the musical piece, and integrating two or more of its parts. Some learning techniques seem effective during all the practice phases (such as segmenting the musical piece into smaller sections so they can be studied separately and practicing with varying tempi to acquire the rhythmic structure of each subsection). As musicians become acquainted with the musical material, the time dedicated to each practice task changes, and the focus shifts mainly toward integrating passages and refining the performance of the whole piece. Similar results were reported by Hallam (2001). When starting to learn a new musical piece, expert musicians (in Hallam’s study) adopted specific strategies for becoming acquainted with the new material, including slow practice and reflection. After the initial phase, they moved to other strategies which allowed them to analyze the musical piece and repeat challenging passages in order to refine and consolidate their recall. From these findings, it seems clear that there are strategies that, in general, are more successful in enhancing musical learning as they focus specifically on the elements of the material that require greater effort to achieve. However, in a metacognitive approach to learning, it is also fundamental to know when a specific strategy may be more useful for accomplishing a musical task.

As is true for expert musicians, music learners need to know many practice strategies and be able to select and apply them effectively. Nielsen (1999, 2004) examined how university music students organized their individual practice, focusing specifically on the selection of learning strategies. Students adopted different cognitive and metacognitive strategies which evolved as they became better acquainted with the piece. They were able not only to select, apply, and monitor the use of learning strategies but also to decide how much time to dedicate to each subtask. These skills can be developed with the help of the teacher, but they also depend on the student’s previous learning experience. These studies have shown that advanced students have a range of different learning strategies that evolves over time, enhancing individual musical practice. These strategies can still be grouped according to the specific task or sub-task being tackled, and this sort of grouping of strategies becomes more evident when examining the habits of less advanced students, as described by Leon-Guerrero (2008). Despite individual preferences, learning strategies in the musical practice of middle school music students can be categorized into different groups (Leon-Guerrero, 2008) according to their prevalent feature and the subtasks in which the strategies may be most effectively applied. Some strategies mainly focus on a few specific musical elements (such as dynamics, tempo, and notes), while others are based on the repetition of difficult beats or passages. In addition, there are strategies that do not directly involve playing, such as fingering or memorizing the rhythm by clapping hands or tapping feet. Finally, there are also strategies that do not seem to be as effective for enhancing practice sessions, such as changing pieces or parts to avoid studying a difficult passage. Inefficacious learning strategies may depend upon incorrect metacognitive beliefs that students have developed as a consequence of particular learning experiences. Indeed, experience may be useful for knowing and experimenting with new strategies, but in some cases, it can be counterproductive. Abushanab and Bishara (2013) found that adult music students were more likely to consider a practice strategy effective if they had experimented with its short-term benefits (pp. 232). More specifically, participants believed (erroneously) that practicing pieces in a fixed order could result in a more stable musical achievement than using a random-order strategy. Knowledge about the learning process and strategies can also be influenced by heuristics. For this reason, novice performers may sometimes exhibit metacognitive beliefs that may appear effective, but, actually, prevent them from enhancing their performance skills. In such cases, novice performers’ metacognitive evaluation of strategies seems to be affected by the first experience they had with the new pieces (Abushanab and Bishara, 2013). For example, studying new musical material in a fixed order at first allows them to learn faster; however, after a retention period, this strategy is less effective for improving their memory of the learned melodies. These findings highlight the importance of prompting music students to reflect on their cognitive activity and adopt a long-term perspective that takes the entire learning process into account.

### Organizing Musical Practice and Evaluating Musical Material and Performance Outcomes

The organization of practice in music includes the evaluation of the musical material and the memorization and retention of the musical pieces: these are key tasks for expert music performers. The ability to evaluate the material to be learned in terms of difficulty and time requirements as well as to monitor one’s own learning process, self-evaluating, and critically revising the achieved results is fundamental for structuring effective practice sessions. Many studies have focused on how expert performers plan and organize their practice (see Table 1). Peynircioglu et al. (2014) found that, in general, musical pieces contain particular cues that expert musicians rely on to make judgments about their performance. These are mainly associated with the intrinsic features of the score, such as the musical syntax and the harmonic pattern. These elements are probably connected to the musical and expressive meaning of the piece, which represents one of the core goals of preparing for performance. The reflective activity of the performer is not limited to the preparatory phase of practice sessions; it is also important as a self-assessment exercise after a musical performance (McPherson and Renwick, 2011), to identify both strong and weak points for improving individual practice.

Planning and evaluation of musical practice are also important for effective memorization. After having analyzed the material to be learned, musicians have to use metamemory (Flavell and Wellman, 1975) for memorizing it. Memorizing frees the performer from the constraint of the score and allows him or her to overcome possible impairments due to emotional and anxiety-related aspects while performing. To understand how good memorization of musical material can be achieved, Ginsborg (2002) examined the memorizing strategies used by expert and non-professional classical singers. Singing requires the application of specific memorization strategies since the songs include both words and music. Ginsborg (2002) found that the singers’ level of expertise did not seem to be directly correlated with their use of more effective memory strategies. What characterized good memory users was a metacognitive and strategic approach to the task. They planned the memorization of the new material and identified in advance the core elements on which they needed to focus. Similar results have been found in instrumental music practice, in which expert musicians need to strategically develop a specific retrieval scheme based on the identification of boundaries in the structure of the musical piece (Williamon and Valentine, 2002). This scheme helps performers memorize the music and, consequently, guides them in the recollection of the piece during the performance. These findings underline the importance of planning and adopting a conscious strategic approach to memorization to improve the mastery of musical pieces.

Considering specifically music tuition, memorizing techniques are rarely addressed explicitly during music lessons due to beliefs that memory strategies are more similar to personalized heuristics than strategic behaviors that can be taught methodically. Students are often required to memorize pieces for specific performance events, but few indications are usually given about how to fulfill this task effectively. In reality, memorization techniques can be successfully taught. Lisboa et al. (2015) studied a young pianist memorizing a new musical piece. Her teacher presented her a learning strategy often used by expert musicians. She was taught to record her thoughts during the memorization process and write them down on the score. The nature of her thoughts evolved during the learning process, showing three different subsequent phases (memorization, developing interpretation, and polishing). The results showed the effectiveness of this strategy, which exceeded the student’s expectations. These findings highlight the need to foster the development of effective memorization strategies during teaching. Memorizing is a complex task, which requires constant monitoring to ensure a successful result, namely the effective recall of the memorized material.

### Enhanced Self-Regulation During Practice Through Metacognitive Skills

Expert musicians can regulate their practice autonomously, becoming their own teachers. The abilities required to regulate individual cognitive processes are related to metacognition (Pintrich et al., 2000), and in general, they seem to be predictors of successful performance (Pintrich and De Groot, 1990). The relationship between metacognition and self-regulation has been widely examined, yet still warrants further theoretical and empirical investigation. Some approaches consider self-regulation a subcomponent of metacognition in music (Benton, 2014), regarding it as the regulation of cognition (Flavell, 1979), while other perspectives seem to adopt the social cognitive approach of Zimmerman (1995), which distinguishes between the ability to regulate individual cognitive processes and a wider concept of self-regulation. From this perspective, self-regulation in music learning is characterized by certain psychological, social, and environmental features (McPherson and Zimmerman, 2011) that influence its development. They include motivation, learning method, time management, behavior, physical environment, metacognitive competences, strategy use, and social support. In a self-regulated practice session, three specific phases can be identified (Jørgensen, 2004; Williamon et al., 2017): (1) the initial thoughts and beliefs based on which task will be planned and structured (forethought); (2) intentional cognitive processes, which direct individual attention and effort (volitional control); (3) the critical reflection and evaluation that follow the accomplishment of the task (self-reflection).

Expert musicians show a high level of metacognitive competence and self-regulation skills (Varela et al., 2016), which are fundamental for allowing them to reach a high level of professionalism. As Hallam (2001) concurs expert musicians use these abilities to assess their own performance, to recognize positive aspects and also critical points for improvement, and to select, apply and monitor learning strategies during musical practice. Each performer adopts a personalized attitude toward musical practice, showing individual preferences in the use of specific strategies, and in the organization of practice time. However, all these different approaches are guided by the need to create more effective and successful individual learning processes. For example, some performers may prefer to start with sight-reading, in order to get acquainted with the whole piece. Others could, at first, adopt non-playing strategies, analyzing the harmonic structure of the piece, and for gaining a deeper understanding. Araújo (2016) examined self-regulated practice strategies in expert musicians and found three main categories of behaviors: organization of practice, management of personal resources, and management of external resources (pp. 283). The first category refers to behaviors that are focused on planning, structuring, and monitoring practice sessions; the second includes awareness and self-reflection about individual characteristics and needs, the learning process, and goals to be achieved; the last category refers to external support received from teachers, colleagues, and audio and video material that can be used to improve performance. The latter highlights the social dimension of self-regulation (McPherson and Zimmerman, 2011).

The key role of self-regulation for expert musicians reflects also on the educational experience of music students. The development of high-level metacognitive knowledge and skills may help students become more competent learners. As reported in Table 1, recently research in music education has focused specifically on examining how music students develop skills for self-regulating their musical practice. Novice players seem to be less effective in structuring their musical learning: they know several practice strategies, but they are not capable of organizing them well to improve their practice (Hallam, 2001). As they gain expertise in music learning, music students develop a self-regulated approach to musical practice, showing a wider range of learning strategies, and increasingly effective ways of selecting and applying them during individual practice. Bartolome (2009) found that young beginner recorder players who experienced positive results in performance showed a high level of metacognitive competence that resulted in self-regulated practice. They had a goal-oriented approach to musical practice, and they effectively used a large set of learning strategies to study the musical pieces they were assigned during music lessons. High-performing pupils put effort into selecting specific learning strategies for maximizing their practice time, focusing specifically on the beats or sections that contained the trickiest passages (pp. 47–48). In this study (Bartolome, 2009), the recorder students were not explicitly instructed in learning strategies, and spontaneously developed a self-regulated attitude; however, the importance of social support (namely, from the parents) in organizing and managing these students’ musical practice was reported and highlighted. In general, students gradually develop autonomous skills for organizing, regulating, and monitoring their learning activity (McPherson and Zimmerman, 2011). For each of the psychological and cognitive components of self-regulation, the music student should gradually move from external regulation (e.g., teacher/expert model) to an intrinsically motivated attitude toward self-regulation.

### Managing Self-Regulation in Collaborative Practice and Performance

The metacognitive features presented until now reflect an individualistic approach to music practice and performance. But performers are also often engaged in collective performances in bands, choirs, orchestras, and ensembles. This implies the need to include also a specific sub-dimension of metacognition for collaborative performance activity, as suggested by Benton (2014). In group music making the individual effort needs to be integrated effectively in the collective performance. For this, performers need to manage a twofold metacognitive process. Not only do they have to regulate and monitor their individual performance, but they also have to consider the performing outcome of the overall group. This also means setting both individual and group goals, and planning individual practice in order to fulfill these kinds of learning goals. Under these learning conditions the individual is part of a community of practice (Schiavio et al., 2018). The need to pursue collective goals requires advanced music students to develop a particular metacognitive flexibility (Gaunt and Westerlund, 2013; Schiavio et al., 2019), for constantly lining up individual performance with the requests of the group, revising, if needed, individual plans for practice. Another key aspect of collaborative practice that can influence the development of performers’ metacognitive competence is related to the social dimension. As reported by Nielsen et al. (2018), interactions with other musicians may affect decisions, and choices about individual practice routine. Performers may engage in collaborative reflection about the most effective strategies for managing musical practice and learning. On the other hand, they may be also stimulated by a competitive climate to strive for the best possible individual performance contribution. Collaborative learning in group music making may have a positive influence for music students too (Hanken, 2016), empowering their individual metacognitive competence through shared experiences, practice strategies and social support.

The research considered so far shows that metacognition in musical performance is a multidimensional construct. In Figure 1 the main aspects concerning the development of metacognition in music performance are summarized from a social-cognitive perspective (e.g., McPherson and Zimmerman, 2011) which acknowledges metacognition as a dimension of self-regulation. In the final section, the educational implications of this research will be discussed and considered in context.

## The Metacognitive Dimension of Music Performance: Educational Implications

In the context of Western classical music, during their training music students must learn how to organize and regulate their learning processes and musical practice to improve their performance, and reach high-level performance outcomes. Since advanced students can typically manage and regulate their musical learning process, it becomes essential to understand how they acquire metacognitive knowledge during their training. Beginner performers, at first, might encounter many difficulties in adopting a strategic learning approach. It may be thought that before one can acquire strategic abilities and reflective thought, it is necessary to reach a specific level of technical skills; however, research findings show that the achievement of musical skills is enhanced if students are simultaneously supported in critically directing, planning, and evaluating their learning experience (Hallam, 2001). More specifically, there are some metacognitive tasks that teachers can present in music lessons for successfully supporting students’ development of metacognitive competence:

(1) Highlight the role of metacognition in music learning and its importance in achieving a self-regulated practice (Bauer, 2008; Colombo and Antonietti, 2017).
(2) Examine students’ practice in order to understand their level of metacognitive competence, for better defining possible educational interventions (Miksza, 2012; Miksza et al., 2018).
(3) Present different learning strategies, and encourage students to adopt them for structuring effective practice sessions (Nielsen, 2004; Bathgate et al., 2012; Hart, 2014).
(4) Underline the importance of planning and organizing both teaching and learning activity, showing effective strategies for practice organization (Hart, 2014).
(5) Offer models of metacognitive and strategic behaviors, in order to help students acquire self-regulated attitudes toward musical learning (Benton, 2013; Gaunt, 2017; Power and Powell, 2018).

In what follows, some indications for fostering students’ development of metacognitive knowledge and skills will be discussed with reference to recent findings in music education research.

### Highlight the Role of Metacognition in Music Learning and Its Importance for Achieving a Self-Regulated Practice

There are several factors that can influence the development of metacognitive skills in young musicians. The most important is represented by students’ awareness of the role of metacognition in music learning. Any educational intervention focused on this dimension requires the active engagement of students, and metacognitive aspects should be explicitly addressed and discussed during music lessons. According to Bauer (2008), understanding the role of metacognitive skills in music learning is the first step in enhancing students’ abilities to recognize critical issues in their performance, find effective solutions and establish achievable learning objectives. Teachers have an important role to play in promoting a metacognitive attitude toward musical learning. However, if students are not explicitly invited to focus on metacognitive features in music lessons, it may become very difficult to enhance their self-regulated learning strategies. This is what Colombo and Antonietti (2017) found when examining multiple cases of teacher-student interactions during music classes. In their study, some piano lessons given by an expert teacher with four adult students (two beginner and two advanced) were video recorded and coded. Although the teacher worked hard to adopt a metacognitive attitude toward music teaching, the students did not recognize this effort, and complained about a lack of metacognitive instructions. Colombo and Antonietti (2017) hypothesized that if students are not aware that the educator was focusing on the metacognitive dimension of learning, they cannot direct their efforts toward working on, and improving their metacognitive skills. So, it appears that teachers need to ensure that their students are aware of the metacognitive approaches being taught during lessons. In Nielsen’s study (Nielsen, 2004), the age of the learners emerged as a key aspect for structuring metacognitive intervention. For example, adult learners usually have already acquired general metacognitive and self-regulation abilities, and music teachers should start with these general abilities but subsequently channel them in a specific musical context. Students’ age is one of the core features affecting metacognitive competence in music performance, as reported in Figure 1. During a lesson, adult beginners may be encouraged to make explicit, and discuss their practice strategies (how and why they chose to practice a particular passage). Adult learners may be also encouraged to make questions about music, for sustaining their interest, and stimulating self-reflection. If the role of metacognition is properly addressed during training, students may be better equipped to monitor their practice, and achieve specific learning goals.

### Examine Students’ Practice in Order to Understand Their Level of Metacognitive Competence, for Better Defining Possible Educational Interventions

If metacognition can be affected by previous experiences, it may be useful for teachers to understand the degree to which their students are able to adopt metacognitive strategies independently and express critical judgments. For this purpose, music educators have to observe critically their students in action during music lessons, and look for emerging strategical elements. Some tools can help the teacher in this task. Miksza (2012) developed a self-report questionnaire to assess self-regulated learning behaviors and attitudes in young music students. This instrument has proven to be a useful support for teachers to help them discuss metacognition and learning processes with their pupils based on what they have learned in their educational experience. In addition, the self-evaluating questionnaire may help educators promptly recognize students with limited metacognitive competencies to support them in improving their planning and self-regulation in musical practice. With adolescent and adult students, it may also be useful to critically discuss and reflect with them about their musical practice. This may not only help the teachers to understand what a student’s metacognitive level is, but also foster a student’s awareness about the metacognitive dimension of his/her musical performance (Miksza et al., 2018).

### Present Different Learning Strategies, and Encourage Students to Adopt Them for Structuring Effective Practice Sessions

Training that specifically addresses metacognition in music classes may be useful for raising students’ awareness of the importance of developing strategic learning behaviors. According to Bathgate et al. (2012), teaching metacognitive strategies and supporting critical self-reflection regarding individual practice can improve performance in adolescent music students. Under this condition, students developed a more effective and self-regulated learning process, which resulted in better evaluations of their performances compared to their peers who did not experiment with a metacognitive teaching method. During lessons, students were invited to pay particular attention to plan their practice session, and to analyze each new piece in terms of its key features and level of difficulty (Bathgate et al., 2012, pp. 405). They were also encouraged to evaluate their performance and identify both positive aspects and areas for improvement. In addition, they were presented with new, effective strategies for studying musical pieces. Surprisingly, in this study, self-efficacy did not differ between metacognitive and non-metacognitive students. This may indicate that in the very first years of music tuition, it is necessary to explicitly address metacognitive knowledge and skills to offer students support in becoming autonomous. However, a different result was found by Nielsen (2004), who examined adult music students. A positive correlation between the use of cognitive and metacognitive strategies and the level of self-efficacy emerged, showing that students with a high sense of efficacy were more likely to adopt strategic behaviors in their musical learning.

These contrasting results may be due to the different ages of the music students in the two studies: adolescents are still developing cognitive and metacognitive abilities in different academic domains, while adult learners, who have a high level of personal, professional, and academic experience, may have already acquired strategic and metacognitive skills, as well as personal awareness of their individual potential, and weaknesses in the learning process. For these reasons, teaching metacognitive strategies should take into account the age and level of music learners. As Colombo and Antonietti (2017) reported, young music students need more support and monitoring from teachers, while for more advanced students, an emphasis on reflection about strategies may be more useful. For example, children in the first stages of music tuition may be guided in analyzing a new musical piece in terms of structure and possible difficulties, identifying the learning goals, and selecting the strategies for the first practice sessions. The teacher may ask them specific questions for fostering self-reflection about these metacognitive features. More advanced students, instead, may be encouraged to discuss the strategies they chose to structure their practice, for example how they worked on the piece, managed their time, or acted upon their self-reflection. This may follow a gradual process where the role of the teacher shifts from instructor to facilitator, fostering the development of students’ autonomy in organizing and managing effectively the learning process. Considering these findings, it emerged that using learning strategies effectively implies knowing several strategies, being able to apply them and recognizing in what situations, and tasks they can be most useful. To foster these abilities, teachers may present students different strategies, and discussing with them their possible benefits in musical practice.

### Underline the Importance of Planning and Organizing Both Teaching and Learning Activity, Showing Effective Strategies for Practice Organization

Not only is metacognition needed to promote a life-long learning approach in musical performance, but metacognitive knowledge and strategies learned in music lessons can also be transferred to other cognitive domains, promoting a general self-regulated approach to learning. To enhance metacognitive strategies in music students, it is not sufficient to present them with different practice techniques, but it is necessary to explain and discuss with students how these techniques can be used and in what tasks they can be applied more effectively. This is the key point on which Hart (2014) based his educational approach for fostering young music students’ metacognitive abilities. In his approach, Hart (2014) recognized the role of deliberate practice in supporting the empowerment of beginner music students’ skills. Music educators should help students organize their practice sessions, select strategies and allocate time resources, guiding the development of pupils’ metacognitive competence. Music teachers should promote reflective activity among their students, encouraging them to evaluate their learning process and results, and to make explicit the strategies they put into action while studying music. For this purpose, it can be useful to suggest the use of specific tools, such as a practice guideline prepared by the teacher for beginner pupils, or a diary for daily practice, where the more advanced students can autonomously take note each day about learning goals, strategies adopted, difficulties encountered, etc. More specifically, Benton (2013) identified three main types of learning tasks that should promote a metacognitive and reflective attitude in music students: reflection about learning processes and outcomes, self-assessment, and “think-aloud” sessions in which pupils make explicit the sequence of actions they planned and acted on to accomplish a musical task.

### Offer Models of Metacognitive and Strategic Behaviors, in Order to Help Students Acquire Self-Regulated Attitudes Toward Musical Learning

When students have become aware of the key role of metacognitive abilities in musical learning, different strategies can be adopted to promote a strategical approach to music education. From this perspective, Power and Powell (2018) investigated the learning process and practice habits of young string players and found that as they develop their expertise, musicians tend to pay increasing attention to the quality of their musical practice. In addition, the results revealed that teachers and educators play an important role as models of musical practice. In general, as indicated in Figure 1, metacognitive learning can benefit from modeling (Benton, 2013; Hart, 2014). During music lessons, teachers can propose examples of metacognitive learning behavior and talk explicitly with their students about how and when to apply specific learning strategies, how to evaluate individual performance, and how practice sessions can be improved. Moreover, teachers should help students recognize their strengths and weaknesses in music learning (Hallam, 2001), for enhancing their practice routine. By acting as mentors (Gaunt, 2017), teachers can help create a positive interpersonal relationship with their pupils. In addition, they can also implement a metacognitive attitude in their own teaching activity by reflecting on each educational situation, monitoring and revising their and their students’ progress, and identifying the best possible solutions to specific students’ needs (Schön, 1983). The teacher, for example, may play with passion a new musical piece, thus showing the students what the final result of their learning effort may be. He or she may also lead by example by encouraging the pupils to be curious to explore new pieces and to question their learning process for reaching the best possible result. Family members can also support the development of metacognitive competence in students (Hart, 2014). This is true especially for very young music pupils. This condition can be promoted by offering supervision during musical practice at home and monitoring children’s homework. To make pupils autonomous, parents’ support should gradually decrease to allow the strengthening of students’ self-regulation skills in musical learning activity.

## Conclusion

Music performance represents the final outcome of a multidimensional process in which musicians acquire the necessary competence for successfully playing and performing new musical pieces. Performing requires constant practice, which, at the highest level of musical expertise, needs to be accurately prepared. Accordingly, to do this, players must develop high competence in planning in advance by self-regulating and assessing their learning activity. In this perspective, metacognition is a core component of musical practice and should be addressed more specifically in music tuition. Different aspects influence the development of metacognitive competence in musical performance (see Figure 1), and they all have to be considered in music lessons. Although interest in this topic is recent, suggestions for practical applications in pedagogical contexts and some possibilities for further research have been highlighted by this review. For example, it may be useful for teachers to assess a new student’s metacognitive abilities and implement approaches to strengthen those skills. This can highlight the key aspects that each teacher should take into account while starting working with a pupil. In addition, another aspect that should be examined more in detail is the relationship between metacognitive competence and self-efficacy in the musical domain and how it evolves during a musician’s training and professional career.

With reference to the educational dimension of music performance, students who have high metacognitive abilities are more effective in structuring their practice and achieving successful outcomes (Nielsen, 1999). If the metacognitive dimension is explicitly addressed in music classes, it can help students become aware of the role of strategic behavior in enhancing their learning process in music (Bauer, 2008). Research findings have shown that the first step in structuring a metacognitive approach in music teaching is raising students’ awareness of the importance of adopting a metacognitive approach in music learning (Colombo and Antonietti, 2017; Power and Powell, 2018). Without such awareness, improving metacognitive and strategical skills seems impossible. Effective practice cannot be considered only in terms of time spent practicing, because quantity may be worthless without quality (Power and Powell, 2018). For this reason, it is essential to direct music students’ attention toward strategical aspects of their learning activity (Hallam, 2001), encouraging them to openly discuss the main features of their cognitive processes as they learn the musical material, and develop musical skills. A self-regulated learning attitude may be achieved if during music lessons, teachers act as a “learning facilitator” (Rogers, 1969; Creech and Hallam, 2017), supporting students in taking a more active role in organizing, and leading their educational experience with the aim of helping them become autonomous learners.

## Author Contributions

EC critically revised and discussed the literature review of the manuscript.

## Conflict of Interest Statement

The authors declare that the research was conducted in the absence of any commercial or financial relationships that could be construed as a potential conflict of interest.
